# Venoms of Heteropteran Insects: A Treasure Trove of Diverse Pharmacological Toolkits

**DOI:** 10.3390/toxins8020043

**Published:** 2016-02-12

**Authors:** Andrew A. Walker, Christiane Weirauch, Bryan G. Fry, Glenn F. King

**Affiliations:** 1 Institute for Molecular Biosciences, The University of Queensland, St Lucia, QLD 4072, Australia; glenn.king@imb.uq.edu.au; 2 Department of Entomology, University of California, Riverside, CA 92521, USA; christiane.weirauch@ucr.edu; 3 School of Biological Sciences, The University of Queensland, St Lucia, QLD 4072, Australia; bgfry@uq.edu.au

**Keywords:** venom, toxin, predation, haematophagy, paralysis, liquefaction, venomics, venom discovery, Heteroptera, true bugs

## Abstract

The piercing-sucking mouthparts of the true bugs (Insecta: Hemiptera: Heteroptera) have allowed diversification from a plant-feeding ancestor into a wide range of trophic strategies that include predation and blood-feeding. Crucial to the success of each of these strategies is the injection of venom. Here we review the current state of knowledge with regard to heteropteran venoms. Predaceous species produce venoms that induce rapid paralysis and liquefaction. These venoms are powerfully insecticidal, and may cause paralysis or death when injected into vertebrates. Disulfide-rich peptides, bioactive phospholipids, small molecules such as *N*,*N*-dimethylaniline and 1,2,5-trithiepane, and toxic enzymes such as phospholipase A_2_, have been reported in predatory venoms. However, the detailed composition and molecular targets of predatory venoms are largely unknown. In contrast, recent research into blood-feeding heteropterans has revealed the structure and function of many protein and non-protein components that facilitate acquisition of blood meals. Blood-feeding venoms lack paralytic or liquefying activity but instead are cocktails of pharmacological modulators that disable the host haemostatic systems simultaneously at multiple points. The multiple ways venom is used by heteropterans suggests that further study will reveal heteropteran venom components with a wide range of bioactivities that may be recruited for use as bioinsecticides, human therapeutics, and pharmacological tools.

## 1. Evolution of Venom Systems in Heteroptera

### 1.1. Introduction: Are Heteropterans Venomous Animals?

**Figure 1:**
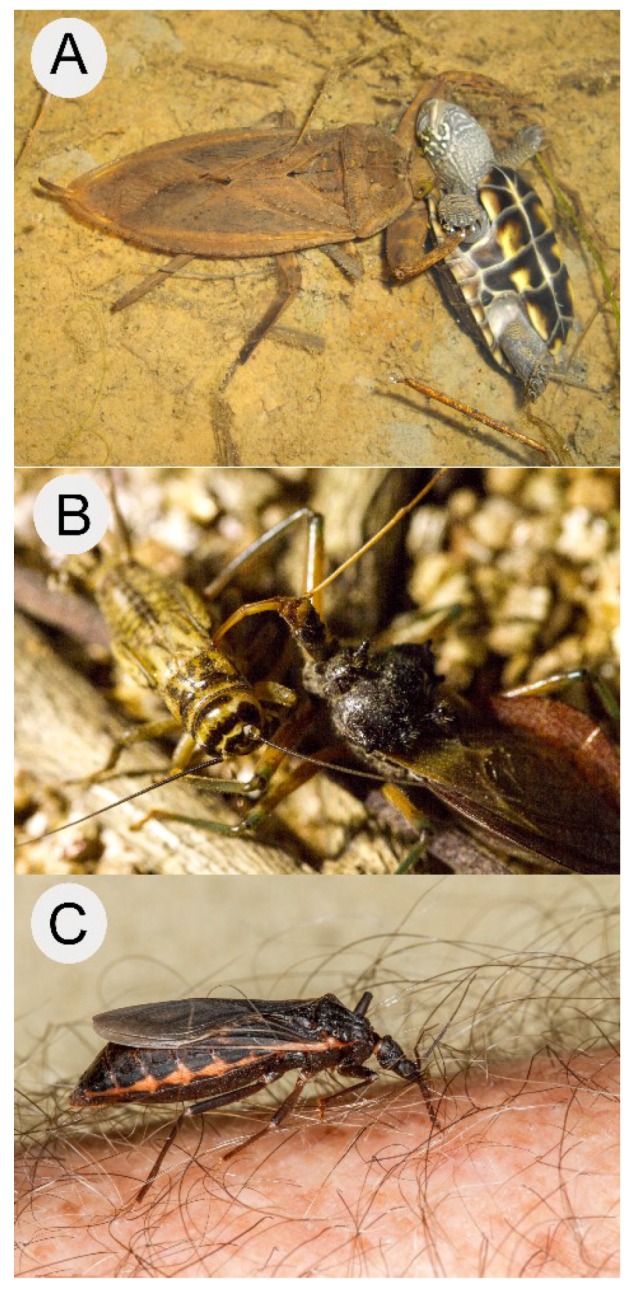
**Venomous heteropterans.** (**A**) An aquatic predaceous heteropteran, the giant water bug *Kirkaldyia deyrolli*, with turtle prey. Photo © Shin-ya Ohba; (**B**) A terrestrial predaceous heteropteran, the assassin bug *Pristhesancus plagipennis*, feeding on a cricket; (**C**) A blood-feeding heteropteran, *Triatoma rubida*, feeding on human blood. Photo © Margy Green.

The suborder Heteroptera or true bugs (Figure 1) are a morphologically and ecologically diverse group of insects within the order Hemiptera. Like the remaining groups of hemipterans (e.g., cicadas and aphids), the true bugs have piercing-and-sucking mouthparts. However, while other hemipterans are exclusively phytophagous, the true bugs have evolved to prey on arthropods and other animals and to become ectoparasites of vertebrates. Central to the evolution of these trophic shifts has been the adaptation of the piercing-and-sucking mouthparts—used by hemipterans to feed on plants—into a sophisticated venom apparatus. Since envenomation first evolved as a trophic strategy in an insect ancestral to present-day heteropterans, they have diversified into 42,000 species in 89 families and seven infraorders. Today, they occupy a wide range of ecosystems and represent one of the most successful radiations of hemimetabolous insects [1,2].

As a result of their widespread distribution, interactions between heteropterans and humans are frequent, diverse, and economically important. Kissing bugs (Reduviidae: Triatominae) bite vertebrates (including humans) to feed on their blood; in the process they spread trypanosomes responsible for Chagas disease—a condition that results in more than 7000 deaths per year and substantial diminution in quality of life for affected individuals [3]—and cause allergic responses including anaphylaxis [4]. Predaceous heteropterans such as assassin bugs (Reduviidae) and backswimmers (also known as water bees, Notonectidae) may bite humans, causing pain, tissue necrosis, numbness, respiratory disturbances, and in extreme cases, death [5,6,7,8,9]. While some plant-feeding heteropterans are agricultural pests that incur billions of dollars in management costs annually, others are valued biocontrol agents in agricultural ecosystems [1,10]. A central feature in each of these relationships between humans and the true bugs is envenomation. Thus, understanding the venom systems of heteropterans is a key feature in understanding both their evolutionary history and present-day management.

We regard all predaceous and blood-feeding heteropterans to be unambiguously venomous under the definition of Fry and colleagues [11]: “Venom is a secretion, produced in a specialised gland in one animal, and delivered to a target animal through the infliction of a wound … a venom must further contain molecules that disrupt normal physiological or biochemical processes so as to facilitate feeding or defense by the producing animal”. Unfortunately, there is a historical tradition among entomologists and venom researchers to confuse or discount the venomous nature of heteropterans. For example, Baptist [12] concludes his classic study of heteropteran labial glands by remarking that “The toxic nature of the saliva of the predaceous forms seems to be incidental to their being of the nature of digestive juices”. The essence of this view, commonly echoed in more recent studies (e.g., [13]), is that true bugs inject digestive enzymes but not neurotoxins. In particular it is supposed they do not inject neurotoxins such as those found in the venoms of spiders, snakes, scorpions, cone snails, centipedes and cnidarians that bind to specific ion channels and receptors in the injected animal to quickly induce paralysis, pain or death [14,15,16,17,18,19]. The roots of this confusion lie in the unique feeding biology of heteropteran predators and a paucity of data on heteropteran venom biochemistry. Since hemipteran mouthparts only permit the uptake of liquid food, liquefaction of prey by extra-oral digestion (EOD) [20,21] is of utmost importance to predaceous heteropterans to maximise nutrient intake (Section 1.2). The production of large quantities of concentrated enzymes to assist in EOD is undoubtedly a key function of the labial glands, which are also the source of venom. However, there is also clear and convincing evidence that heteropterans inject into their prey not only digestive enzymes but also neurotoxins and other pharmacological modulators.

The venom of predaceous species is not only used to digest prey, but for defense (by causing pain when injected into a potential predator) and to rapidly immobilise and kill prey. Edwards [6] noted more than half a century ago that venoms from assassin bugs such as *Rhynocoris carmelita* and *Platymeris rhadamanthus* are able to induce paralysis both potently (bugs being able to paralyse prey hundreds of times larger than themselves) and quickly (over a time scale of seconds). Some assassins such as *Holotrichius innesi* are even able to kill vertebrates by a single envenomation, which induces respiratory paralysis after 15–30 s in mice [9]. These results strongly suggest the presence of neurotoxins. Schmidt [22] and Zlotkin [23] provide thoughtful discussions on the case for neurotoxins in true bug venoms, noting that the high potency of the venoms and the reversibility of their toxic effects with washing [6,9,24] argues in favour of the presence of neurotoxins. The most direct demonstration of neurotoxins in the venoms of predaceous heteropterans to date is perhaps the discovery that assassin bug venom contains peptides that adopt the inhibitor cystine knot (ICK) structure which is widespread in venom neurotoxins from other animals [25,26]. Neurotoxic activity of these peptides has been demonstrated, revealing that the venoms of predaceous heteropterans and other venomous taxa have evolved along strongly convergent lines (Section 2.2.3) [27,28]. To date, evidence of neurotoxic activity has been obtained from the venoms of just a few families, but the vast majority of heteropteran venoms have never been investigated using techniques capable of identifying and characterising neurotoxins. Neurotoxins may therefore be widespread in the venoms of predaceous true bugs.

In contrast to the predaceous bugs, blood-feeding heteropterans do not require their hosts to be paralysed. Instead, they need to circumvent the haemostatic and sensory processes of the host that normally prevent loss of blood and detection of parasites. Due to their status as ectoparasites on vertebrates and vectors of blood-borne human diseases, the venoms of blood-feeding heteropterans—especially Triatominae and to a lesser degree Cimicidae—have been characterised in much greater detail than those of their predaceous counterparts (Section 2.3). These studies have revealed a multitude of bioactive molecules that specifically target host haemostatic and defence systems, and which have evolved with a high degree of convergence to venom toxins from other blood-feeding animals [29].

### 1.2. Evolution of the Heteropteran Venom Apparatus

**Figure 2:**
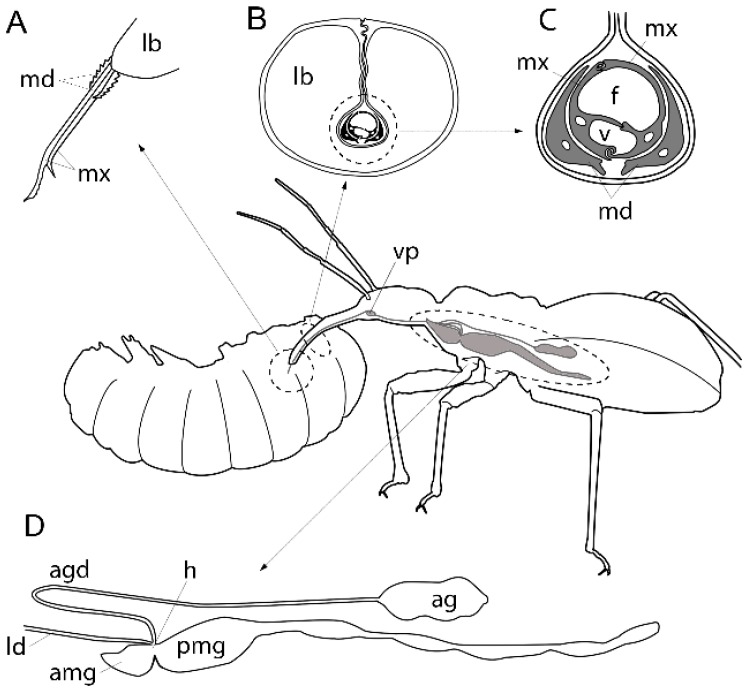
The heteropteran venom apparatus. The central figure shows the position of key anatomical structures involved in envenomation, in this case for prey capture by a reduviid. For clarity, although lateral ducts from venom glands on each side of the body would merge into a common duct shortly before reaching the venom pump (vp), only the left venom gland complex is illustrated. (**A**) Insertion of mouthparts into the prey, showing mandibular (md) and maxillary (mx) stylets emerging from the tip of the labium (lb); (**B**) Cross-section of the proboscis showing the labium surrounding the stylet bundle; (**C**) Enlarged cross-section of mandibular and maxillary stylets. Note the asymmetry of the maxillary stylets and separate food (f) and venom (v) canals. The small hole in each stylet indicates the position of a nerve process; (**D**) Labial gland complex showing anterior lobe of the main gland (amg), posterior lobe of the main gland (pmg) and accessory gland (ag). The lateral duct (ld) leading to the salivary pump and proboscis, and the accessory gland duct (agd) connecting to the accessory gland meet the main gland at the hilus (h). Adapted from Cobben [30], Cohen [31], and Smith [32].

All Hemiptera—whether predaceous, haematophagous or phytophagous—feed through a structure called a proboscis or rostrum (Figure 2). The proboscis consists of highly derived mouthparts that enable it to function like a double-barrelled syringe [30,31,32]. The bulk of the visible proboscis is formed by the labium, greatly elongated and concave dorsally (with proboscis extended) so that it forms a hollow tube or sheath. Within this sheath lie the mandibles and maxillae, also greatly elongated into structures known as stylets (Figure 2a–c). The mandibular stylets lie outside the maxillary pair, do not interlock, and are often tipped with barbs or serrated edges. The inner maxillary stylets are asymmetric and (with very few exceptions) interlock to form two separate fluid canals: the food canal dorsally, and the salivary canal ventrally (Figure 2c). A devoted muscle-driven pump within the head powers transmission of fluid through each canal. The salivary pump—situated at or close to the junction of the maxillary salivary canal with the two ducts from the labial gland complex on each side of the body—pumps fluid from the labial glands into the food source.

The most common arrangement of the labial glands consists of a main secretory gland with 2–4 lobes, which may extend anteriorly into the head and posteriorly into the abdomen, and an accessory gland (typically located in a more posterior and medial position, often in close apposition to the gut; Figure 2d) [12,33,34]. The paired main glands are connected to the salivary pump via lateral and common salivary ducts, with the accessory gland being connected to the main gland through an additional duct. Numerous variations to this structure occur, even within a single subfamily [12,35,36]. The anterior and posterior lobes of the main gland, and the ducts leading to the accessory gland and the venom/salivary pump, are connected at a complex junction called the hilus. The hilus incorporates an outer and inner mixing chamber that are separated by muscle-controlled valves from each other, from the main gland, and from the efferent duct to the salivary pump [12,35,37]. This anatomical arrangement probably allows the animal to inject the contents of each of the lobes of the main gland and the accessory gland separately.

The main gland typically consists of a single layer of columnar secretory epithelium arranged in a sack-like structure surrounding a large glandular lumen. The columnar cells show extensive endoplasmic reticulum and they are usually observed to be filled with dense secretory granules, consistent with them being the major site of protein production and secretion via a merocrine mechanism [12,35]. The secretory cells are ensheathed in a basal lamina containing muscle fibres, which receive innervation from the hypocerebral ganglion [12]. The accessory gland is usually observed to contain a watery secretion, is well-oxygenated by a special tracheal supply (suggesting high metabolic turnover), but is not innervated. It is usually postulated to have a role in transporting water from the haemolymph to supply the labial glands [38], but it also has a secretory role in some species [39,40]. Though the mechanism of water transport is unknown, some experiments suggest that haemolymph proteins and other solutes may be co-transported into the gland lumen [41].

In phytophagous heteropterans and non-heteropteran hemipterans, the labial glands secrete substances that facilitate feeding by breaking down plant tissue, evading host plant defences, and producing salivary sheaths [42]. In predaceous and blood-feeding heteropterans, the labial glands secrete venom that facilitates feeding by paralysing and liquefying prey through EOD or that combat host haemostatic systems. Radiolabelling experiments have shown that heteropteran venoms originate not in the gut (as in many other arthropods that practice EOD) but solely in the labial glands [20,31]. For venomous species, we propose that the terms *venom canal* and *venom pump* are appropriate to use in place of *salivary canal* and *salivary pump*. Although the exact function of each labial gland structure is currently uncertain, we also use the term *venom gland* interchangeably with *salivary gland* and *labial gland.*

Predaceous heteropterans ambush, stalk or actively chase their prey. Some species have specialised structures or behaviours that assist in prey capture, including raptorial forelegs and/or adhesive pads (see Section 2.1.1 and Section 2.2.1). As the prey or host becomes close, the proboscis is extended. Envenomation typically occurs in a swift strike in which the mandibular and maxillary stylets penetrate the food source and venom is injected, sometimes accompanied by grasping or gripping with the forelegs or fore- and mid-legs. Within this quick movement the sheath-like labium, its tip covered with chemo- and mechanoreceptors, is pressed to the surface of the food source; the pointed tips of the mandibular stylets extend to cut into the prey and anchor it to the predator [30,31,32]; and the maxillary stylets extend into the prey, injecting venom. Once venom injection has induced paralysis or death [6,9], feeding typically occurs over a period of several minutes to several hours. During this process the stylets may extend deep into the prey, distributing liquefying venom, macerating the prey, and sucking up fluid food [31]. These actions are facilitated by the supremely agile nature of the stylets, which are innervated structures able to turn up to 180° to access deep cavities of the legs and antennae [31,32]. In blood-feeders such as *Rhodnius prolixus*, venom is injected continuously throughout feeding [32]. As the maxillary stylets probe tissue for blood vessels, they sample the surrounding material. The presence of ATP (an abundant component of red blood cells) induces them to gorge [43].

Phytophagous heteropterans produce different secretory products within different parts of the labial gland complex [44,45], and some experiments indicate that the glands of predatory heteropterans are also functionally compartmentalised. Haridass and Ananthakrishnan [35] prepared separate homogenates from the main gland anterior lobe, main gland posterior lobe, and accessory glands of two predaceous reduviids (*Peirates affinis* and *Haematorrhophus nigroviolaceus*). Injecting homogenates of main gland anterior lobes into prey insects resulted in rapid paralysis, whereas injection of posterior lobe homogenate resulted in no immediate effects but death after several hours. Injection of the accessory gland homogenate had no effect. These authors concluded that the anterior and posterior lobes are specialised to secrete neurotoxins and digestive enzymes, respectively. Other authors have reported similar findings, albeit usually with less drastic differences between the two lobes of the main gland [46,47]. Only Edwards [6] observed no difference between the effects of homogenates from anterior and posterior lobes of *P. rhadamanthus* applied to cockroach heart-dorsum preparations. This contrasting finding may represent either a taxon-specific difference or an effect of dosing. Thus, more detailed studies are required to clarify if assassin bug labial glands, and those of other predaceous heteropterans, are functionally compartmentalised like the venom glands of cone snails [48] and centipedes [49].

### 1.3. Diversification of Trophic Strategies in the Heteropteran Radiation

**Figure 3:**
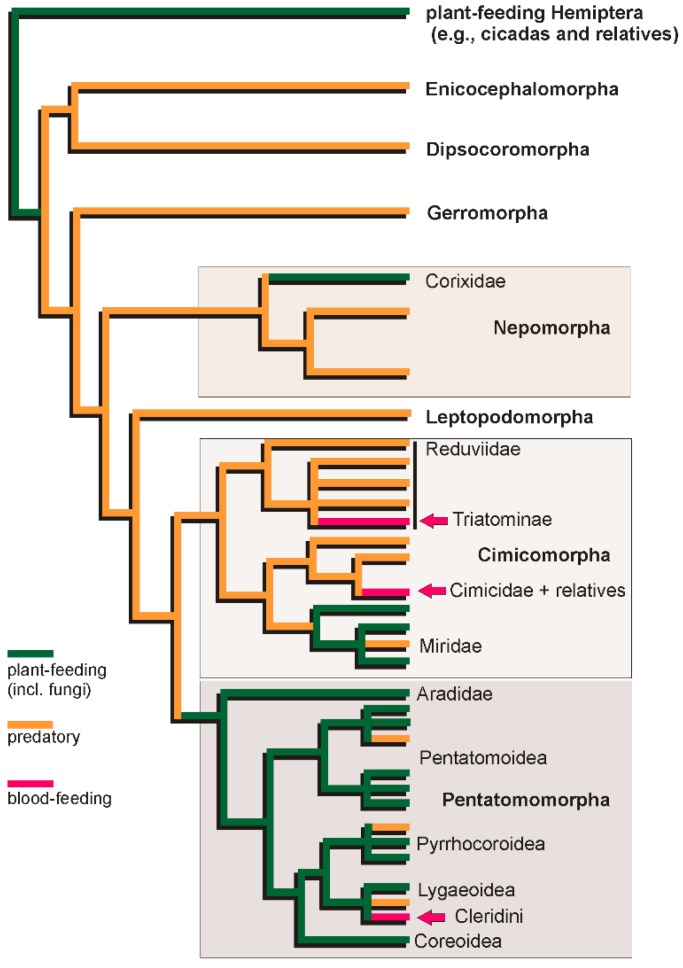
Phylogram showing trophic strategies (phytophagous, predatory, and blood-feeding) across Heteroptera. Phylogenies simplified and modified from Wang and colleagues [51], Schuh and colleagues [52], and Hua and colleagues [53].

The highly derived mouthparts and labial glands of hemipterans—originally adaptations to feed on plants—are powerful preadaptations for the development of envenomation. Envenomation is practised even by some obligate plant-feeding Sternorrhyncha (soldier aphids; [50]), but it is the heteropterans, whose ancestors switched to a predatory trophic strategy, that the vast majority of venomous hemipterans belong (Figure 3).

Our understanding of the evolution of the different feeding types in Heteroptera has been hampered by the lack of well-supported phylogenetic hypotheses across the suborder, although some general patterns are well-established. Phylogenetic resolution and support amongst the infraorders Enicocephalomorpha, Dipsocoromorpha, Gerromorpha and Nepomorpha that we here refer to as “Lower Heteroptera” [2,51,54] are currently ambiguous. Nevertheless, all four lineages are almost exclusively composed of predators, lending support to the hypothesis that the last common ancestor of Heteroptera was likely also predaceous [55,56]. This predatory life-style appears to have been retained in the last common ancestors of Leptopodomorpha (shore bugs end relatives) and Cimicomorpha (assassin bugs, bed bugs, plant bugs and relatives), but a transition to mycophagy and/or phytophagy occurred in the common ancestor of Pentatomomorpha (stink bugs and relatives), with a reversal to predation in some pentatomomorphan groups. The largest clade of predominantly phytophagous heteropterans, the Miroidea (plant bugs and relatives), represents a secondary transition to plant-feeding within Cimicomorpha. In addition, obligate blood-feeding has evolved at least three times independently within Cimicomorpha (Cimicidae and Polyctenidae; triatomine Reduviidae) and Pentatomomorpha (Rhyparochromidae: Cleradini and Udeocorini) [56].

The radiation of venomous heteropterans into diverse trophic strategies has resulted in different selection pressures acting on venom toxins. Extant true bugs have venoms that are adapted to the way they hunt, feed and defend themselves. In addition to this “passive” evolution of venom toxins, evolutionary innovations in venom systems may be active drivers of evolution, allowing the invasion of new niches and habitats. For example, the highly sophisticated molecular machinery underlying the hypermutation of cone snail venom peptides [57,58] has likely driven the recent explosive radiation of the marine snail genus *Conus* and the adoption of new trophic niches such as feeding on fish [59]. Therefore, studying heteropteran venom systems and venom biochemistry may assist our understanding of their evolutionary radiation. In the next section, we review the major groups of venomous heteropterans and explore how venom pharmacologies have been adapted to facilitate particular feeding strategies.

## 2. Diversification of Venom Pharmacology in the Evolution of Heteroptera

### 2.1. Aquatic and Semi-Aquatic Hunters: Nepomorpha, Gerromorpha and Leptopodomorpha

#### 2.1.1. Habitat and Prey Range

Several groups of true bugs are associated with water. The Nepomorpha (true water bugs), Gerromorpha (semiaquatic bugs) and Leptopodomorpha (shore bugs) account for most of the species not contained within the speciose terrestrial infraorders Cimicomorpha and Pentatomomorpha. As their name suggests, the true water bugs spend most of their lives submerged, with the exception of the Gelastocoridae (toad bugs) and Ochteroidea (velvety shore bugs), which like the Leptopodomorpha, occupy freshwater shore zones and some terrestrial habitats [56]. Gerromorpha such as pond skaters, that hunt suspended upon the surface tension of the water, are one of the few insect groups that successfully exploits marine environments.

The giant water bugs and water scorpions (Nepoidea = Belostomatidae + Nepidae) are ambush predators that typically await prey in submerged vegetation [56]. Raptorial forelegs occur in Gerromorpha and the nepomorphan families Belostomatidae, Gelastocoridae, Nepidae, Naucoridae and Notonectidae. Plant-feeding is rare in aquatic and semi-aquatic Heteroptera, having evolved apparently only in the water boatmen (Corixidae) [56]. The most common prey items consumed are aquatic crustaceans, insects, snails, worms, tadpoles, and small fish. Various aquatic heteropterans are regarded as biocontrol agents in natural and disturbed aquatic environments, especially for larvae of disease-vectoring mosquitoes [60,61,62,63]. Some giant water bugs (Belostomatidae) grow to be very large (10–12 cm) and have been recorded killing vertebrates including frogs, turtles, snakes and birds [64,65,66,67]. From a pharmacological point of view, this prey range is interesting as it suggests belostomatid venom may contain toxins selected for bioactivity against vertebrate molecular targets.

#### 2.1.2. Activity and Composition of Nepomorphan Venoms

Aquatic and semi-aquatic heteropterans inject venom to immobilise and liquefy prey. Most information regarding the venoms of the water-associated groups is from Belostomatidae, Nepidae and Notonectidae, while essentially nothing has been recorded of the venoms of Gerromorpha or Leptopodomorpha. Humans bitten by aquatic water bugs experience pronounced pain, swelling, vasodilation and sometimes numbness [7] while invertebrates and small vertebrates bitten by belostomatids or injected with their venom are typically paralysed after several minutes [24,68]. Venom gland extracts equivalent to 1% of the glands of the creeping water bug *Naucoris cimicoides* dissolved in 100 μL saline result in immediate cessation of beating of the cockroach heart-dorsum preparation [6]. Venom from the giant water bug *Belostoma anurum* similarly prevents pumping of the heart-dorsum of the triatomine *R. prolixus*, and reduces the amplitude of rat sciatic nerve compound action potentials; both these effects were reversible and mediated by low-molecular weight compounds (<5 kDa) that are not destroyed by proteases [69]. Belostomatid venom injected into guinea pig hearts causes bradychardia and an increase in resting tension not mediated through effects on cholinergic receptors or Ca^2+^ channels [70]. Picado [24] found that bites from *Belostoma delpontei* paralyse frogs within several minutes. Diluted whole-head homogenates produce a reversible paralysis in fish 15 min after being introduced to the water in which they were swimming.

The detailed composition of belostomatid venom is unknown, but electrophoresis of the venom reveals many protein components ranging from low molecular weight peptides (<5 kDa) up to proteins ~55 kDa in size [39,70,71]. Enzymatic assays (Table 1) have revealed phospholipase A_2_, hyaluronidase, protease, amylase, esterase, α-glucosidase, glucosaminidase, invertase, lipase, nuclease, phosphatase and phosphohydrolase activities [39,68,71,72]. Of these, phospholipase A_2_ and hyaluronidase are of particular interest because they have been convergently recruited into phylogenetically diverse animal venoms [11]. Venom phospholipase A_2_ toxins bind with high affinity to sites on synaptic clefts and muscles, where they exert toxic effects through enzymatic cleavage of membrane glycerophospholipids to yield lysophospholipids and free fatty acids (both potent physiological modulators); they may also act through non-enzymatic mechanisms [73,74]. In contrast, hyaluronidase has been proposed to be a “spreading factor” that increases the permeability of tissue to other toxins [75]. Water scorpion (Nepidae) venom has not been examined in terms of its protein content or bioactivity, but a transcriptomic study of salivary glands from *Ranatra chinensis* revealed many transcripts encoding proteins with homology to proteases, acid phosphatases, apyrases, dipeptidylpeptidases IV, hyaluronidases, and prophenyloxidases [76]. Aside from protein components, Silva-Cardoso and colleagues [68] reported a rich lipidic content of venom from the giant water bug *B. anurum*, which was found to contain 88% lipid and only 12% protein. These authors focused on the presence of lysophosphatidylcholine (lysPC) in water bug venom. LysPC is a product of the degradation of phosphatidylcholine by phospholipase A_2_, and has been shown to have a variety of toxic effects including inhibition of neurotransmitter release and vasodilation [77,78]. LysPC accounted for approximately 1.8% of the venom, and venom-purified lysPC and unpurified venom containing an equivalent amount of lysPC were found to cause paralysis when injected into zebrafish (*Danio rerio*).

Aside from the small number of studies on the venom of aquatic bugs, an additional complication arises from the complexity of the secretory glands in this group. As well as the normal complement of main labial glands with anterior and posterior lobes and accessory glands, the belostomatids, nepids and gelastocorids contain additional secretory glands—termed by various authors “cephalic”, “maxillary”, or “poison” glands—that lie ventrally in the head or anterior thorax [79,80]. These glands are unconnected to the labial/venom glands, and they open not into the venom canal of the proboscis but into two ventrolateral slits near the base of the proboscis. Their precise role is unclear, but insects expel a “milky” secretion from these glands when threatened or handled that is potently insecticidal [79] and produces a burning sensation if applied to a puncture in human skin [80]. This secretion may perform a defensive function. Thus, it is important that future studies clarify if the “white and viscous” lipid-rich venom characterised by Silva-Cardoso and colleagues [68] corresponds to the “milky” secretion produced in the cephalic glands, or to the venom injected into prey that is produced by the labial glands.

### 2.2. Assassin’s Creed: Terrestrial Predators in Cimicomorpha and Pentatomomorpha

#### 2.2.1. Efficient Predation through Envenomation, Prey-Capture Organs and Dietary Specialisation

Many species of the higher Heteroptera are efficient terrestrial predators. Envenomation, which allows swift killing of prey many times larger in size than the predator—together with quick lifecycles, active hunting behaviours on plants and trees, and dietary preferences that are either broad or actively favour plant-damaging prey—make many predatory heteropterans excellent biocontrol agents in agricultural systems compared to other venomous animals. For example, single big-eyed bugs (*Geocoris punctipes*) or assassin bugs (*Pristhesancus plagipennis*) are respectively capable of consuming 1600 spider mites or 220 cotton bollworms through the nymphal stages alone [81,82]. Numerous methods for the mass rearing of heteropteran predators have been developed using artificial diets [83], and representatives of the Anthocoridae, Nabidae, Miridae, Pentatomidae, and Reduviidae are in active commercial use [10,83].

**Figure 4:**
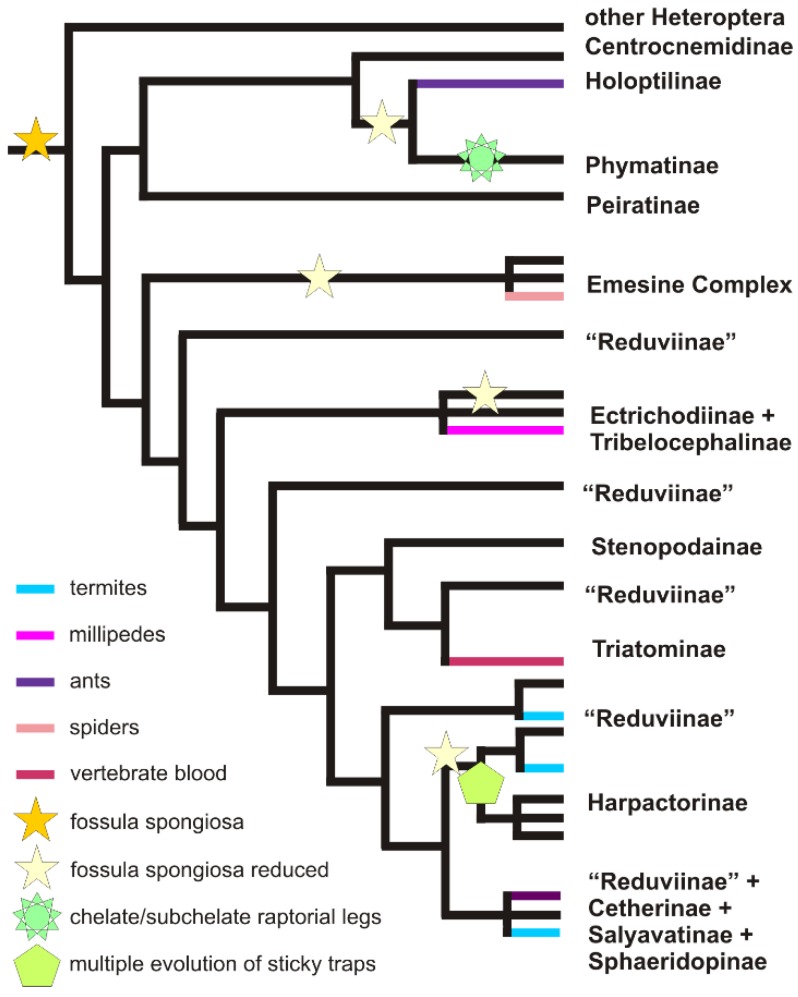
Prey specialisations and raptorial leg modifications within Reduviidae. Modified and simplified from Hwang and Weirauch [112], Gordon and Weirauch [127], and Zhang and colleagues [106].

Besides envenomation, predation is assisted by a variety of morphological and behavioural adaptations. One such adaptation is the fossula spongiosa, an adhesive structure on the fore and sometimes middle tibia of many cimicomorphans used for gripping the prey [101]. This structure was recently shown to have been part of the ancestral raptorial leg of Reduviidae and was lost multiple times during the radiation of assassin bugs (Figure 4) [52]. Various lineages of reduviids subsequently evolved alternate methods for holding on to prey [102]. For example, in a group of harpactorine Reduviidae that includes the large and widespread genus *Zelus* (leafhopper bugs)*,* bioadhesives are secreted from glands on the fore- and mid-legs [103,104]. The resin bugs (tribes Apiomerini, Diaspidiini, and Ectinoderini, also in the subfamily Harpactorinae) collect sticky plant resins which are applied to the forelegs to assist prey capture [105]—a behaviour that has recently been shown to have arisen independently in each group [106]. Raptorial forelegs with enlarged femora and/or heavy armature on the ventral surfaces of foretibia and forefemur occur in other lineages of Reduviidae, including the ambush bugs (Phymatinae) and thread-legged bugs (Emesinae) [107,108]. In venomous groups such as scorpions, coevolution of venoms and grasping forelegs (pincers) has occurred such that the two systems are complementary [109]. A similar trend may occur in Heteroptera, as assassin bugs lacking a fossula spongiosa have been reported to take less time to paralyse prey [110,111].

Venom toxicity and specificity is likely also to vary with prey preference. Many heteropteran predators are generalists, feeding on a wide range of prey, and generalism is probably the ancestral condition [112]. Some heteropterans display more restrictive prey choices, such as prostemmatine Nabidae on other Heteroptera [113] or isometopine Miridae on scale insects [114]. Many of the prey specialists currently documented occur in Reduviidae (Figure 4). Prey specialisation has frequently been accompanied by the evolution of specialised morphological and behavioural adaptations. For example, some lineages of the thread-legged bugs (Reduviidae: Emesinae) are facultatively or obligately associated with spider-webs, where they either steal prey captured in the spider’s webs or prey on the spiders themselves [115,116]. Araneophagic emesine bugs have evolved convergently to araneophagic spiders [117] in developing a form of aggressive mimicry in which plucking of web strands is used to produce vibrations that manipulate the behaviour of the prey spider [118,119,120,121]. The feather-legged bugs (Holoptilinae) specialise on ants and employ both visual and chemical luring strategies [122,123]. Juveniles attract prey by waving their hind legs, which are covered in a cluster of brush-like setae. Despite the dangerous nature of their prey—which are many times larger and possess their own insecticidal venom—the bugs only strike when actually grasped in the ant’s mandibles [124]. Adult feather-legged bugs possess an even more powerful luring system, secreting a pheromone from a ventral organ called a trichrome [125,126]. This pheromone attracts, apparently mesmerises, and may paralyse ants. After typically a long period of time crawling around and over the assassin, the ants are eventually killed when they attach their jaws to the trichrome itself—a position placing the vulnerable area at the base of its head directly below the assassin’s feeding proboscis. Prey conservatism on specific groups of termites has been documented using forensic and crowd-sourcing methods for assassin bugs in the subfamilies Salyavatinae and Sphaeridopinae [127]. Members of one genus in the subfamily Salyavatinae have been shown to “fish” for their prey with just-fed-upon termite carcasses to catch workers assiduously trying to retrieve the bodies of their dead [128]. Prey specialisations have evolved independently to those listed above in the Ectrichodiinae for millipedes [129]; in Cetherinae [130], the harpactorine genera *Tegea* [131] and *Micrauchenus* [132], and the reduviine genus *Leogorrus* [130] for termites; in some members of the reduviine *Acanthaspis* clade [133] for ants; and in the harpactorine genera *Scipinia* and *Nagusta* for social jumping spiders [134].

While a small amount is known about the morphological and behavioural adaptations accompanying prey specialisation in heteropterans, almost nothing is known about the accompanying changes in venom composition. The multiple independent instances of prey specialisation among the assassin bugs suggests they are a potential source of prey-specific toxins, which are of interest for biotechnology [135]. Further study of assassin bug venom may also offer an opportunity to contribute to discussions of how the evolution of prey specialisation and capture organs such as raptorial forelegs affects venom composition [110,136,137,138,139].

#### 2.2.2. Physiological Effect of Venoms of Terrestrial Predaceous Heteropterans

##### Effects of venom on invertebrates 

Predaceous cimicomorphans and pentatomomorphans use their venoms primarily for immobilising, killing, and liquefying invertebrate prey. A key effect of venoms is rapid paralysis, which has been reported after envenomation by members of the Reduviidae [6,9,35,140], asopine Pentatomidae [141], predaceous Miridae [85], and predaceous Lygaeidae [142], and which may be much more widespread. The insecticidal activities of two reduviine assassin bug venoms have been quantified as LD_50_ values of (dry weight of venom) 2 mg/kg (*H. innesi* venom injected into larvae of the fly *Sarcophaga argyrostoma*) [9] or 10.25 mg/kg (*P. rhadamanthus* venom injected into the cockroach *P. americana*) [6]. Insecticidal activity has also been demonstrated in the venoms of harpactorine (*Rhynocoris* sp.) and peiratine (*Catamiarus brevipennis*) assassin bugs, but with doses that are likely substantially higher (2–9 μL of crude venom per gram of insect tissue) [46,111,140,143]. While Edwards [6] found that *P. rhadamanthus* venom had no effect when applied topically or orally to flies and cockroaches, both Sahayaraj and Muthukumar [143] and Sahayaraj and Vinothkanna [143] found that adding venom of *Rhynocoris* sp. diluted approximately 1:1000 to the feed of caterpillars over 96 h was sufficient to cause death.

The effects of reduviid venom have also been studied on isolated insect nerve preparations. Edwards [6] found that adding undiluted *P. rhadamanthus* venom to cockroach heart-dorsum preparation induces violent contracture of the heart and surrounding musculature, immediate cessation of beating, and a slow relaxation of excitable tissue. Diluted venom, down to one part in a million, resulted in initial increases in heart rate followed by irregular beating and eventually cessation. Similar results were obtained with venom from the predatory reduviids *Rhynocoris carmelita* (Harpactorinae) and *Reduvius personatus* (Reduviinae). In contrast, the venoms of blood-feeding reduviids (*Triatoma protracta* and *Rhodnius prolixus*) showed no effect, and venoms from lygaeid bugs with phytophagous or facultatively predaceous lifestyles showed much weaker modulation of heart-dorsum pumping at much higher doses. Venom from *P. rhadamanthus* induced burst firing followed by non-responsiveness in cockroach giant fibres, loss of conduction in non-synaptic preparations from the locust *Schistocerca gregaria*, and general lytic activity on all body tissues. Edwards concluded that the major action of assassin bug venom is non-specific disruption of lipid membranes, but the high potency of the venom and the observation that heart-dorsum preparations from *P. rhadamanthus* are immune to the bug’s own venom suggest the additional presence of neurotoxins.

##### Effects of venom on vertebrates

In addition to prey capture and feeding, many species use venom for defence and will envenomate humans if handled. The minute pirate bugs (Anthocoridae) owe their name to the disproportionate amount of pain their bites induce relative to their small size (1–5 mm). Some assassin bugs such as the reduviine *P. rhadamanthus* also spit venom defensively [144]. This behaviour is apparently directed at vertebrate predators and has arisen convergently to similar behaviour in snakes, spiders and wasps [145,146]. Most heteropteran envenomations are not medically important for humans. The most common symptoms are pain, redness, and swelling. However, envenomations by some species induce respiratory disturbances and tissue necrosis [6]. At least one species, the “afrur” (*H. innesi*), a reduviine assassin bug occurring in the Middle East, is suspected of being responsible for several human fatalities by envenomation [5].

Zerachia and colleagues [9,147] characterised the effect of *H. innesi* venom on mammalian tissues. Surprisingly, venom from this bug is more toxic to mice (LD_50_ 1 mg/kg) than to fly larvae (LD_50_ 2 mg/kg). Toxicity testing of size-fractionated venom indicated the factors causing insect toxicity co-purified with cytolytic activity while the factors causing mammalian toxicity eluted later [23]. A single envenomation is capable of killing a mouse in 15–30 s, apparently through respiratory paralysis [9] and a cytolytic component capable of lysing red blood cells is also present [147]. Sub-lethal doses of venom induce paralysis and local haemorrhage when injected intramuscularly, nocifensive behaviour when injected intraperitoneally, and reduced blood pressure and peripherally-restricted haemorrhage when injected intravenously [9]. Venom applied to rabbit cornea induced lacrimation, blinking and hyperaemia, while guinea pigs inhaling aerosols containing venom died due to respiratory paralysis. Low concentrations of venom caused slow contraction of guinea pig ileum preparations and blocked acetylcholine-induced contractions in a reversible manner. Injection of venom into the femoral artery of a cat completely and reversibly blocked contraction of the gastrocnemius muscle in response to sciatic nerve stimulation, similar to the action of the nicotinic acetylcholine receptor antagonist curare [9]. These effects suggest the presence of an acetylcholine receptor antagonist and perhaps sodium channel modulators. Thus, venoms from assassin bugs in general, and *H. innesi* in particular, are likely to contain neurotoxic molecules capable of modulating the activity of channels and receptors in the mammalian nervous system [148].

#### 2.2.3. Composition of Venoms from Terrestrial Predators.

Much of what is known about venoms from terrestrial predaceous heteropterans is from one family, the assassin bugs (reviewed by [149]). Edwards [6] recorded the pH of *P. rhadamanthus* venom as 6.8, which is similar to the pH of labial gland lumens (6.0–6.8; [12]). Sahayaraj [88] observed a decrease in *R. marginatus* venom pH from 7.4 one day after feeding to 5.1 after seven days starvation. Infrared spectra of dried *R. marginatus* venom suggests it consists predominantly of protein [88]. The protein content of assassin bug venom depends on species, sex, time since emergence or feeding, and the gland lobe in which venom is produced [6,46,88,150,151]. Sahayaraj and colleagues [151] examined venoms from bugs fed the previous day, finding protein concentrations of 24 and 54 mg/mL respectively for males and females of *R. marginatus*, and 28 and 86 mg/mL for males and females of *C. brevipennis*. Ambrose and Maran [150] and Maran [46] compared the concentration of venom extracted from each lobe of the main glands of *Acanthaspis pedestris* and *Rhynocoris* sp. respectively (without homogenisation of gland tissue) after feeding and over each day of prey deprivation. Interestingly, the contents of the anterior lobe were consistently more concentrated than those of the posterior lobe by roughly a factor of two. The concentration of venom from both lobes increased steadily up to eight days starvation. In *A. pedestris*, the protein concentration increased from 155 to 224 mg/mL for the anterior lobe and from 78 to 124 mg/mL for the posterior lobe. Maximum concentrations of 414 mg/mL and 201 mg/mL were reached for the anterior and posterior lobes, respectively, of *R. kumarii*. Sahayaraj and colleagues [88] recorded protein concentrations up to 420 μg/mg wet venom. Thus, assassin bug venom concentrations are in the same range reported for other animal venoms [152,153].

**Figure 5:**
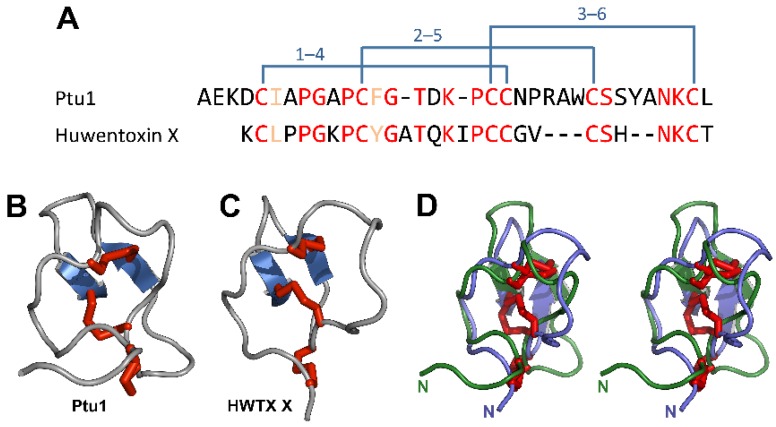
Similarity of ICK toxins from spiders and assassin bugs. (**A**) Alignment of the amino acid sequences of Ptu1 from venom of the assassin bug *Peirates turpis* [27] and huwentoxin-X (HWTX-X) from venom of the tarantula *Haplopelma schmidti* [156]. Identical and similar residues are highlighted in red and orange, respectively, and the disulfide-bond architecture is shown above the sequence alignment. The peptide sequences are remarkably similar (~50% identity) and both toxins target Ca_V_2.2 channels; (**B**, **C**) Schematic of the 3D structures of Ptu1 (PDB 1I26) and HWTX-X (PDB 1Y29). Disulfide bonds and β-strands are coloured red and blue, respectively; (**D**) Stereo view of an overlay of the structures of Ptu1 (green) and HWTX-X (blue). The *N*-termini are labelled.

The major classes of toxins present in venoms of predaceous heteropterans are unknown. However, the studies of Corzo and colleagues [28] and Bernard and colleagues [27] revealed that some assassin bug venoms contain disulfide-rich peptide toxins with a high degree of structural convergence to venom peptides from cone snails, scorpions and spiders. In Corzo’s study, venoms from three assassin bugs (*Agriosphodrus dohrni* and *Isyndus obscurus*, both Harpactorinae; and *Peirates turpis*, Peiratinae) were fractionated via liquid chromatography. Fractions selected based on their mass spectra were then sequenced via Edman degradation. These methods revealed a homologous peptide in all three bugs that contains 34–36 amino acid residues with a conserved pattern of six cysteine residues (Figure 5) and structural similarity to the ω-conotoxins produced by cone snails. The peptide from *P. turpis*, Ptu1, was then synthesised in quantity using Fmoc solid phase peptide synthesis. Like the ω-conotoxins, synthetic Ptu1 was shown to inhibit *N*-type voltage-gated calcium channels (Ca_V_2.2) [28] and adopt the ICK fold [27]. A DALI search [154] reveals that the 10 closest structural homologs of Ptu1 are actually ICK toxins from spider venom. The closest sequence/structural homolog is Huwentoxin-X from the tarantula *Haplopelma schmidti* that, in a striking example of convergent evolution, also targets Ca_V_2.2 (Figure 5). Peptides in similar mass ranges have been observed in venoms from genus *Rhynocoris* using MALDI-TOF mass spectrometry (MS) [88,155] and may be widespread among reduviids and other predatory heteropterans.

In contrast to these findings, the recent study of Martínez and colleagues [157] found that venom of the predatory stinkbug *Podisus nigrispinus* is toxic to the caterpillar *Anticarsia gemmatalis* because it contains small molecule toxins including *N*,*N*-dimethylaniline and 1,2,5-trithiepane. Insecticidal activity was concentrated in ether extracts rather than aqueous extracts of venom glands, and toxicity was only mildly affected by treatment with either protease inhibitor cocktails or proteinase K. These results suggested that venom toxicity was due to the presence of hydrophobic non-proteinaceous molecules, which were subsequently identified by gas chromatography-MS. Thus, asopine pentatomids such as *P. nigrispinus* may rely less on peptide and protein toxins for subduing prey compared to their reduviid relations. This difference in toxin composition may be related to the transition to phytophagy and then reversion to predation as a trophic strategy in the ancestors of Asopinae (Section 1.3).

In addition to neurotoxic peptides and non-protein small molecules, venoms from cimicomorphans and pentatomomorphans contain larger protein components that underlie numerous enzymatic activities (Table 1). Sahayaraj and colleagues [88] observed assassin bug (*R. marginatus*) venom proteins to occur mainly in the range 2–37 kDa, while Habibi and colleagues [158] observed that most venom proteins in spiny soldier bug venom (Pentatomidae: Asopinae) are in the range 15–90 kDa. Phospholipase A_2_ and hyaluronidase, which are present in the venoms of aquatic heteropterans and many other venomous animals (Section 2.1.2), are prominent in predatory species but not phytophagous species (Table 1).

Other enzymatic activities reported are protease, esterase, lipase, phosphatase, invertase and trehalase (Table 1). These may be most easily ascribed a function in liquefaction, but may also contribute to toxicity. By far the most commonly reported enzymatic activity is proteolytic. In almost all species the primary protease activity is trypsin-like (as determined by an alkaline pH optimum, substrate and inhibitor specificity) [6,84,85,86,90,94,159]. Cohen [84] observed three different proteins with trypsin-like activity in the salivary glands of *Zelus renardii* (Harpactorinae), the most prominent of which had a molecular mass around 27 kDa. Frequently, lesser amounts of chymotrypsin-like endopeptidase and amino- and carboxypeptidases are present (Table 1). Collagenase activity has also been reported in venom of the predatory bug *Podisus nigrispinus* [160]. Some omnivorous heteropterans feature enzymes that break down plant-specific biopolymers, such as amylase and pectinase. Surprisingly, low levels of amylase are also detected in obligate predators (Table 1) which may represent relic activity, previously unsuspected omnivory, or the ability to break down the gut contents of herbivorous prey.

The only transcriptomic study of a venom gland from a terrestrial heteropteran predator comes from a minute pirate bug (family Anthocoridae). Baek [161] made a subtractive cDNA library enriched for transcripts expressed at higher levels in the venom glands compared to whole body of the minute pirate bug *Orius laevigatus*. The most abundant transcripts in this library encoded proteases, haemolysins, carbonic anhydrases, DNAses, lipases, cathepsin B1, alkaline phosphatase, CUB domain proteins, bacterial-permeability-increasing protein and many unknown proteins. Thus, anthocorid venom may have cytolytic activity by virtue of membrane-disrupting proteins such as haemolysins and bacterial-permeability-increasing protein.

### 2.3. The Blood Feeders

#### 2.3.1. Convergent Evolution of Blood-Feeding in Heteroptera

Some true bugs have built further on the evolutionary success of predation, becoming ectoparasites feeding on vertebrate blood. The evolution of blood-feeding may have occurred in the nests of birds and mammals, where predaceous bugs attracted by the plentiful invertebrate prey were easily tempted to experiment with haematophagy by the close proximity of helpless vertebrate juveniles [112,162]. Obligate blood-feeding has evolved in Heteroptera several times independently: at least once and perhaps twice or more among the kissing bugs (reduviid subfamily Triatominae) [163,164], and at least once in the ancestors of the bed bugs (Cimicidae) and the closely related bat bugs (Polyctenidae). Several other heteropteran groups have been reported to feed on blood to various degrees. Some rhypochromine Rhypochromidae (tribes Cleradini and Udeocorini) practise both facultative blood-feeding from vertebrates and kleptohaematophagy through feeding on engorged haematophages [165,166,167,168]. Minute pirate bugs (Anthocoridae) and non-triatomine assassin bugs are occasionally recorded to feed from vertebrates and are sometimes considered facultative blood-feeders (e.g., [164]). For vertebrates, including humans, bites from blood-feeding insects represent not only an annoyance and nutritive drain but also the potential of anaphylactic reactions and contraction of blood-borne diseases. The most important disease vectors in Heteroptera are the kissing bugs that transmit *Trypanosoma cruzi*, the causative agent of Chagas disease [169]. Because of their status as human disease vectors, the venoms of blood-feeding heteropterans have been studied in much greater detail than those of their predaceous counterparts, and excellent recent reviews are available [29,170,171,172,173,174,175]. This section therefore aims not at comprehensive review but to compare and contrast the venoms of blood-feeding and predaceous heteropterans.

#### 2.3.2. Venoms of Haematophagous Heteroptera

Early studies on venom glands from blood-feeding heteropterans established that both the high activity of digestive enzymes and the potent paralytic activity that characterise venom from predaceous heteropterans were lacking [6,12]. Among blood-feeding bugs, paralysing activity has only been reported in the context of facultative entomophagy [176]. These results are consistent with the fundamentally different activities required of venom used to acquire blood meals compared to venom used to subdue and liquefy prey. Since vertebrate hosts are typically many orders of magnitude larger than haematophagous bugs, remaining undetected during feeding presents a better strategy than inducing systemic paralysis. Moreover, already-liquid blood does not require enzymatic liquefaction, as do the tissues of invertebrates. Instead, these blood-feeders have to overcome the sophisticated and highly redundant haemostatic defense systems that protect host tissues from blood loss, invasion and injury—the most important of which are platelet aggregation, vasoconstriction, blood coagulation, and the pain and itch pathways [175].

To achieve this feat, heteropteran blood-feeding bugs have evolved a complex cocktail of pharmacological agents that affect many different components of the haemostatic system at once, simultaneously dilating blood vessels [163,177,178,179] and inhibiting platelet aggregation [180,181,182,183] and coagulation [184,185,186,187]. Triatomine venom is also able to inhibit voltage-gated sodium channels [188], which may account for its anaesthetic effect [189,190]. Laboratory trials have shown that even alert humans are usually unable to detect triatomine bites if they cannot see the bug [191]. In contrast, without the ability to inject venom (for example when the labial gland complex is ablated), triatomines experience considerable difficulty in obtaining a blood meal even from restrained hosts [192].

Over the last 25 years, the molecular basis of the venom’s action has been studied through two complementary strategies. First, bioactive components have been characterised functionally after purification from crude venom or recombinant expression (Table 2). Secondly, venom protein sequences have been discovered using venom-gland transcriptomics and/or proteomics [239,240,241,242,243,244,245,246,247,248,249,250,251,252]. These studies have revealed much convergent evolution, both between heteropteran lineages with separate evolutionary origins of blood-feeding, and between heteropteran blood-feeders and blood-feeders from other animal taxa. For example, apyrases, enzymes that prevent ADP-induced platelet aggregation by degrading ADP, occur in a wide range of blood-feeding animals including triatomine bugs (Reduviidae: Triatominae) and the bedbug *Cimex lectularius* (Cimicidae) [29,253]. However, while triatomine apyrases belong to the same 5' nucleotidase family as mosquito venom apyrases do [227,254], bedbug apyrases belong to an unrelated protein family that has been convergently recruited in the venom of biting sand flies [238,255]. Likewise, most venoms used to facilitate blood-feeding have vasodilatory activity, which may be achieved through a variety of mechanisms and targets [175]. In both the bedbug *C. lectularius* and triatomine bugs of the genus *Rhodnius* the main vasodilatory components are a group of nitric oxide (NO) donating proteins, the nitrophorins [159,186,256,257]. Perhaps surprisingly, triatomine bugs in the genus *Triatoma* do not produce nitrophorins and their venom exerts vasodilatory effects through other mechanisms [163]. These observations may support the hypothesis of multiple origins of blood-feeding within Triatominae [162]. Although the triatomine bugs in genus *Rhodnius* and the bedbug *C. lectularius* have convergently evolved venom nitrophorins, *Rhodnius* nitrophorins belong to the lipocalin family whereas *C. lectularius* nitrophorins are descended from an inositol phosphate phosphatase [236]. Many other protein families present have been convergently recruited in venoms from blood-feeding and/or predaceous animals, including the odorant-binding family, cysteine rich secreted protein (CRiSP)/antigen-5 family, and the Kazal domain family [175].

Aside from convergence in function, the evolution of blood-feeding venom reveals radiation and divergence that has allowed closely related proteins to diversify to perform many different functions. For example, lipocalins are proteins that typically have roles in lipid binding, but which have evolved to fulfil an astonishing array of functions in triatomine venom—including donation and sequestration of diverse ligands and inhibition of various host haemostatic proteins (Table 2). Lipocalin-family proteins frequently make up the majority of venom proteins by both number of sequences and abundance [256,257,258]. Some lipocalin family proteins are multifunctional, such as *R. prolixus* nitrophorin-2 (prolixin-S), which acts as a NO donor, binds histamine with greater affinity than human receptors, and binds to factor IX/IXa and thereby inhibits activation of coagulation pathways [186,203,205,207].

While highly adapted to blood-feeding, triatomines are nevertheless descended from predaceous reduviids (most likely in the *Zelurus* group that also includes Stenopodainae and some Reduviinae) [108,259]. Some venom components hint at this evolutionary past and may constitute relict activity. For example, trialysin is a 22 kDa pore-forming protein from *Triatoma infestans* venom that lyses bacterial, protozoan and mammalian cells [229]. However, its occurrence in triatomine venom is of unknown significance since it occurs at concentrations not sufficient to lyse red blood cells, and triatomines in any case store red blood cells in the gut for several weeks before lysis occurs in the posterior midgut. Thus, Amino and colleagues [229] hypothesised that trialysin serves an antiparasitic role by defending the venom glands from microorganisms. Trialysin-like proteins may however contribute to the cytolytic activity that is a hallmark of venoms from predaceous reduviids [6]. Other components are notable due to their absence in triatomine venom. For example, Corzo and colleagues [28] found cystine-rich peptides that are conserved between venoms of the distantly related reduviid subfamilies Peiratinae and Harpactorinae, indicating that the last common ancestor of the Higher Reduviidae—which also includes Triatominae [102,112]—possessed such peptides. However, no evidence of related sequences have to our knowledge been detected in triatomine venom glands, despite deep sequencing (e.g., [248]). The apparent loss of this class of toxins may be consistent with its common occurrence in venoms used to facilitate predation, but comparative rarity in blood-feeding venoms [26].

## 3. Future Directions

The venom systems of true bugs are unique, reflecting their unique biology. Further study of their venom will add to our understanding of the evolution of Heteroptera, facilitate efforts to develop heteropterans as efficient biocontrol agents, and contribute to management of the effects of heteropteran envenomations and disease vectoring. The study of neglected animal venoms such as those of heteropterans will also decrease the taxon bias in our current understanding of venoms and venom evolution. In addition, venoms are recognised to be potent sources of bioactive compounds that may be recruited for use as novel bioinsecticides, therapeutic drugs and pharmacological tools [148,260]. The wide range of trophic strategies, prey types and the use of venom defensively suggests that heteropteran venoms may contain a “treasure trove” of molecules with diverse bioactivities. However, little is known about most true bug venoms. This is especially true of predaceous heteropterans, which should form one focus of future work in this area.

The molecular characterisation of venoms from small insects has become increasingly feasible due to advances in technologies such as next-generation sequencing and mass spectrometry. A logical first avenue of approach would be to examine the evolution of protein toxins in Heteroptera by studying venom proteomes from representatives of the major and more common neglected predaceous groups, especially Belostomatidae, Nepidae, Notonectidae, Reduviidae, Anthocoridae, Asopinae, predaceous members of the Miridae, Lygaeidae and Pyrrhocoridae, and predaceous and blood-feeding forms of the Rhypochromidae. Studies combining transcriptomics of venom glands with proteomics of secreted venom are to be favoured, as these provide unambiguous and detailed information on protein toxins that is unbiased by reliance on homology to known protein toxins. In addition, studies of this type may allow subsequent production of toxins by recombinant expression [261] or peptide synthesis [262] in quantities large enough for functional characterisation. Many heteropterans can be induced to expel venom by electrical stimulation [151,263] or the application of muscarinic acetylcholine receptor agonists such as pilocarpine [38,39]. Where venom cannot be extracted by these methods, proteomics of salivary gland homogenates (e.g., [245]) may be an alternative approach to study venom composition.

A complementary approach to transcriptomic/proteomic studies will be functional studies elucidating the physiological actions, active components and molecular targets of predaceous heteropteran venoms. Toxicity studies of crude and fractionated venoms have the potential to tell us which toxin classes—including small non-protein molecules, linear and disulfide-rich peptides, and larger proteins—are most important for prey paralysis, death and liquefaction. To understand their natural function, it is also desirable to screen heteropteran venoms for bioactivity against as many molecular targets as practicable. Targets present within their natural prey should be prioritised, especially those targeted by other venomous animals and known to induce paralysis, such as voltage-gated sodium, calcium and potassium channels, and acetylcholine and glutamate receptors [264]. For those species that inject venom defensively (possibly encompassing most predaceous heteropterans), it will also be of interest to screen venoms for agonists of nociceptive channels capable of inducing pain and aversion that exist in their natural predators, such as transient receptor potential (TRP) channels [265,266] and acid-sensing ion channels (ASICs) [267].

In further characterising the mechanisms and targets of heteropteran venoms, the use of fractionated venoms is desirable, as components with generalised toxicity (e.g., pore-forming proteins) may mask specific activity (e.g., inhibition of ion channels) both *in vivo* and in cell-based assays *in vitro*. An important consideration in any biodiscovery work is the evolutionary history leading to present-day heteropteran predators. Because some predatory lineages may have independent origins through trophic switching events (e.g., asopine Pentatomidae and isometopine Miridae, both of which are likely derived from plant-feeding ancestors; Section 1.3), a primary guide in considering target species selection and the framing of biological questions should be a detailed understanding of heteropteran phylogeny [2].

Aside from “bottom-up” venom discovery work, Heteroptera offers the opportunity to frame “top-down” questions pertaining to evolution of venom systems. For example, the family Reduviidae contains multiple independent origins of prey specialists descended from generalist ancestors (Section 2.2.1). What effect does prey specialisation have on venom composition in terms of number of components, synonymous *versus* non-synonymous mutations in proteinaceous toxins, and in the prey specificity of the toxins employed? Do predatory lineages separated by trophic switching events differ markedly in their active components, or have the same classes of toxins been recruited multiple times? How does the presence of prey capture specialisations such as raptorial forearms or fossula spongiosa affect the toxicity and composition of venom? Due to its unique evolutionary history, Heteroptera may be an ideal model taxon in which to investigate these questions.

## 4. Conclusions

Heteroptera is a speciose and diverse group of insects. The basic body plan of ancestral plant-feeding hemipterans, featuring piercing and sucking mouthparts, a well-developed and complex secretory apparatus, and a dedicated delivery channel for saliva through the maxillary stylets, constitutes a powerful morphological preadaptation for envenomation.

The switch from a plant-feeding ancestor to a predaceous true bug likely occurred in the last common ancestor of all Heteroptera and the predatory life-style was retained in most lineages, although secondary transitions to phytophagy occurred, especially in the Pentatomomorpha and Cimicomorpha, with subsequent reversals to predation. Today, venomous heteropterans occupy a wide range of habitats including aquatic, sub-aquatic, marine, terrestrial, arboreal, and cosmopolitan. They range from only a few mm long as adults in the case of minute pirate bugs to behemoths of the insect world such as giant water bugs, which may measure over 10 cm. Most predatory heteropterans are generalist feeders on invertebrates, but prey specialisation has evolved multiple times, often in concert with complex hunting strategies. Larger species, especially from the aquatic groups, feed on vertebrates including fish, frogs, snakes, turtles and birds. In addition to prey capture, most predatory heteropterans use venom defensively as a deterrent against predators. Several heteropteran groups have eschewed predatory lifestyles in favour of stealing blood from vertebrates, a habit to with they are now highly adapted.

Concomitant with these diverse lifestyles, heteropteran venoms have evolved to have drastically different physiological effects. The venoms of predaceous heteropterans induce rapid paralysis and liquefaction. The active components producing these effects include neurotoxic disulfide-rich peptides, biologically active phospholipids, cytolytic agents and enzymes. However, there are few detailed studies on the venom of predaceous heteropterans, and the relative importance of these components and their evolution across the heteropteran phylogeny is unclear. In the case of blood-feeders, many bioactive components have been identified and characterised that target vertebrate haemostatic and sensory systems, especially from Triatominae. We anticipate that further studies on heteropteran venoms will yield bioactive molecules with a range of biological activities proportionate to the wide range of trophic strategies used by these insects. Thus, heteropteran venoms may represent a treasure-trove of molecules with utility in biotechnology, medicine, and as pharmacological tools.

## Figures and Tables

**Table 1:** Enzymes detected in venoms of predaceous heteropterans.

Infraorder	Family	Species	Feeding Strategy ^a^	Phospholipase A_2_	Hyaluronidase	Protease				Lipase	Esterase	Invertase	Nuclease	Acid phosphatase	Alkaline phosphatase	Amylase	Pectinase	Refs.
						Trypsin-like	Chymotrypsin-like	Aminopeptidase	Carboxypeptidase									
Nepo-morpha	Belostomatidae	*Lethocerus *sp.^b^	P		yes	yes	yes			yes	yes		yes	yes	yes	no		[39,71]
	Belostomatidae	*Belostoma *sp.^b^	P	yes		yes	yes			yes	?	no		yes	yes	?		[39,68,72]
	Nepidae	*Ranatra elongata*	P			yes				no	no	yes				no		[72]
Cimicomorpha	Reduviidae	*Platymeris rhadamanthus*	P	yes	yes	yes				no	no							[6,42]
	Reduviidae	*Zelus renardii*	P	strong		strong	yes	weak	weak							weak		[31,84,85]
	Reduviidae	*Sinea confusa*	P	strong		strong										weak		[31,85]
	Reduviidae	*Rhynocoris marginatus^c^*	P	yes	yes	strong		weak		yes	yes	yes		yes	yes	yes		[86,87,88]
	Reduviidae	*Catamiarus brevipennis*	P			yes				yes		yes				yes		[87]
	Anthocoridae	*Orius insidiosus*	P			yes										yes		[89]
	Nabidae	*Nabis alternatus*	P,(H)	yes		yes										?		[31,85]
	Miridae	*Deraeocoris* sp.	P	yes		yes	yes										?	[85,90]
	Miridae	*Lygus *sp.	H,(P)	?	no	yes	yes									strong	yes	[85,91,92,93,94]
	Miridae	*Creontiades dilutus*	H,(P)			weak	yes										yes	[95,96]
Pentatom-omorpha	Pentatomidae	*Podisus *sp.^d^	P	?		yes								yes		yes		[31,85,97,98,99]
	Pentatomidae	*Andrallus spinidens*	P			yes	yes	yes	yes									[100]
	Geocoridae	*Geocoris punctipes*	P,(H)	yes		yes				yes						?		[31,85,92]

^a^ P = predator, H = herbivore, brackets indicate facultative feeding. ^b^ Swart and colleagues [39] found additional enzymatic activities in belostomatid venoms including glucosidase, *N*-acetylglucosamidase, and leucine arylamidase. ^c^ Sahayaraj and colleagues [88] also found trehalase activity in *Rhynocoris marginatus* venom. ^d^ Fialho and colleagues also found collagenase and cathepsin-l-like activity in the venom glands of *Podisus nigrispinus*. “Strong” or “weak” indicate activity strength as determined in the original studies while “yes” and “no” indicate simple presence or absence. A question mark indicates conflicting results between studies or species differences within a genus.

**Table 2:** Bioactive components in the venoms of blood-feeding heteropterans.

Molecule	Protein Family/Molecule Class	Species	Physiological Function ^a^	Molecular Target	Reference
Nitric oxide	Gas	*R. prolixus, C. lectularius*	V, PAI	Activates guanylate cyclase	[193,194]
Lysophosphatidylcholine	Lipid	*Rhodnius prolixus*	PAI, other	Unknown	[195]
Nitrophorins 1–4	Lipocalin	*Rhodnius prolixus*	V, PAI, AI	NO donor, also binds histamine	[159,193,196,197,198,199,200,201,202]
Nitrophorin-2 (Prolixin)	Lipocalin	*Rhodnius prolixus*	AC, V, PAI, AI	Additionally inhibits Tenase complex	[186,203,204,205,206,207]
Nitrophorin-7	Lipocalin	*Rhodnius prolixus*	AC, V, PAI, AI	Additionally binds anionic phospholipids to prevent activation of clotting factors and platelets	[208,209,210,211,212]
Amine Binding Protein	Lipocalin	*Rhodnius prolixus*	V	Binds serotonin and norepinephrine	[213,214]
Triabin	Lipocalin	*Triatoma pallidipennis*	AC	Inhibits activation of thrombin	[185,215,216]
Palladipin	Lipocalin	*Triatoma pallidipennis*	PAI	Collagen-induced PAI, mechanism unknown	[181,217,218]
*Rhodnius* Platelet Aggregation Inhibitor 1	Lipocalin	*Rhodnius prolixus*	PAI	ADP-induced PAI by binding to ADP	[219,220]
Triplatin	Lipocalin	*Triatoma infestans*	V, anti-NET	Binds thromboxane A_2_ and prostaglandin F_2__α_	[221,222,223]
Triafestin-1, -2	Lipocalin	*Triatoma infestans*	AC	Inhibits reciprocal activation of Factor XII, prekallikrein	[224]
Dipetalodipin	Lipocalin	*Dipetalogaster maxima*	V, anti-NET	Binds thromboxane A_2_ and various eicosanoids	[180,222]
Dimiconin	Lipocalin	*Triatoma dimidiata*	AC	Inhibits activation of Factor XII	[225]
Antigen-5	Antigen-5	*D. maxima, T. infestans*	PAI	Collagen-induced PAI by scavenging free radicals	[226]
Apyrase (Triatomine type)	5′ Nucleotidase	*Triatoma infestans*	PAI	Degrades ADP	[227,228]
Trialysin	Trialysin	*Triatoma infestans*	Antimicrobial	Pore formation	[229,230,231]
Protease	Trypsin-like	*T. infestans, Panstrongylus megistus*	AC, unknown	Degrades fibrin nets, other unknown function?	[232,233]
Inositol Phosphatase	Inositol phosphatase	*Rhodnius prolixus*	Unknown	Phosphatidylinositol	[234]
Procalin	Lipocalin	*Triatoma protracta*	Allergen	Unknown	[235]
Nitrophorin (*Cimex* type)	Inositol phosphatase	*Cimex lectularius*	V, PAI	NO donor	[236,237]
Apyrase (*Cimex* type)	Apyrase (*Cimex* type)	*Cimex lectularius*	PAI	Degrades ADP	[183,238]

^a^ PAI = platelet aggregation inhibitor; V = vasodilator; AC = anticoagulant; NET = neutrophil extracellular trap; AI = anti-inflammatory; PA = platelet aggregation.
